# A Simple Method for Decreasing the Liquid Junction Potential in a Flow-through-Type Differential pH Sensor Probe Consisting of pH-FETs by Exerting Spatiotemporal Control of the Liquid Junction

**DOI:** 10.3390/s150407898

**Published:** 2015-04-01

**Authors:** Akira Yamada, Satoshi Mohri, Michihiro Nakamura, Keiji Naruse

**Affiliations:** 1 Department of Mechanical Engineering, Faculty of Engineering, Aichi Institute of Technology, 1247 Yachigusa, Yakusa Cho, Toyota City, Aichi Prefecture 470-0392, Japan; 2 Cardiovascular Physiology, Graduate School of Medicine, Dentistry and Pharmaceutical Sciences, Okayama University, 2-5-1 Shikata-cho, Kita-ku, Okayama Prefecture 700-8558, Japan; E-Mails: smohri@med.kawasaki-m.ac.jp (S.M.); michinak@mx4.kct.ne.jp (M.N.); knaruse@md.okayama-u.ac.jp (K.N.); 3 First Department of Physiology, Kawasaki Medical School, 577 Matsushima, Kurashiki-City, Okayama Prefecture 701-0192, Japan

**Keywords:** pH, ISFET, pH-FET, differential pH sensor probe, liquid junction potential

## Abstract

The liquid junction potential (LJP), the phenomenon that occurs when two electrolyte solutions of different composition come into contact, prevents accurate measurements in potentiometry. The effect of the LJP is usually remarkable in measurements of diluted solutions with low buffering capacities or low ion concentrations. Our group has constructed a simple method to eliminate the LJP by exerting spatiotemporal control of a liquid junction (LJ) formed between two solutions, a sample solution and a baseline solution (BLS), in a flow-through-type differential pH sensor probe. The method was contrived based on microfluidics. The sensor probe is a differential measurement system composed of two ion-sensitive field-effect transistors (ISFETs) and one Ag/AgCl electrode. With our new method, the border region of the sample solution and BLS is vibrated in order to mix solutions and suppress the overshoot after the sample solution is suctioned into the sensor probe. Compared to the conventional method without vibration, our method shortened the settling time from over two min to 15 s and reduced the measurement error by 86% to within 0.060 pH. This new method will be useful for improving the response characteristics and decreasing the measurement error of many apparatuses that use LJs.

## 1. Introduction

Solutions are often described in terms of their pH, a common index of acidity or alkalinity used in many fields such as biochemistry, medicine, and the environmental sciences. An accurate measurement of pH values is essential to the application of science and technology. Reliable pH values are usually obtained by potentiometric measurement using glass electrodes [1] or ion-sensitive field-effect transistors (ISFETs) [2,3]. When two solutions come into contact, the liquid junction potential (LJP) [4] between them becomes a major source of error in these measurements. Many investigations over the years have sought to overcome the error caused by the LJP [5], but none have completely succeeded.

When two electrolytic solutions of different compositions come into contact, the difference in the ion activities of the two aspects generates an LJP [4,5]. Nernst [6,7], Planck [8,9], and Henderson [10,11] were the first investigators to study the phenomenon from a theoretical perspective. Nernst [7] and Negbauer [12] also studied the phenomenon experimentally, and they were followed by MacInnes [13] and Scatchard [14]. Various techniques have been devised to minimize the influence of the LJP on the accuracy of pH measurements. A salt bridge with a concentrated solution has long been used to reduce the LJP [15,16,17,18,19,20]. The salt bridge mechanism reduces the LJP substantially when a saturated KCl solution comes into contact with a sample solution [5,21,22]. Yet several millivolts of LJP remain for the measurement of diluted aqueous solutions with low electrolytic concentrations, which causes measurement errors [23,24]. The LJP can rise well above negligible levels if the diluted sample solutions have low buffering capacities or low ion concentrations, such as environmental water or distilled water. The LJP also varies over time, and the degree of the LJP depends on both the shape and form of the liquid junction (LJ) device and the ion composition of the sample solution [25]. New types of salt bridges using water-immiscible ionic liquids have recently been developed as alternatives to conventional KCl salt bridges [5,26,27]. These methods are suitable for measuring many diluted solutions, as they often succeed in near-perfect removal of the LJPs and avoid the contamination of sample solutions from the leakage of saturated KCl solution. KCl salt bridges such as the ceramic plug type or double-junction type leak KCl solution into the sample solution during measurement, thereby contaminating the sample solutions and possibly changing the pH values. Ionic liquid salt bridges are less useful, however, for measuring sample solutions containing hydrophobic ions, as the LJP fluctuates during measurement [26]. The fluctuating LJP limits the utility of these methods for the measurement of sample solutions containing detergents, heavy metal ions, or organic solvents. One type of reference electrode recently studied was composed of carbon nanotubes and polyacrylate membrane [28]; another was made from an ionic liquid doped membrane and three-dimensionally ordered macroporous carbon [29]. Much earlier, LJ devices of many types, shapes, and forms were developed for precise and stable measurements [30]. Dynamic junctions such as flowing junctions [13,25,31,32,33], continuous mixture junctions, and constrained diffusion junctions [30] provide more precise measurements than static junctions such as free diffusion junctions, as the former decrease LJPs. Back in 1920, Lamb *et al.* reported that the stability and accuracy of measurement can only be secured by both preventing the ageing effect and constructing a fresh junction on the LJ [25]. Flowing junctions easily keep LJPs constant and capable of securing high measurement accuracy [25]. A device with a dynamic junction would be impractical, as it would necessarily be large and therefore impossible to insert into integrated glass electrodes or other sensors. In our own work we have found it too challenging, structurally, to achieve a scaled down dynamic junction capable of fitting inside a practical small sensor probe. We expect microfluidics-based technologies [34] to help us downsize the device and improve the measurement characteristics.

Our group has constructed a flow-through-type differential sensor probe using a semiconductor-based pH-sensitive field-effect transistor (pH-FET) [35,36,37]. The micro-sized sensor probe responds quickly and is capable of measuring small sample volumes. The differential sensor system consists of two pH-FETs and an Ag/AgCl pseudo reference electrode designed to works together with a baseline solution (BLS) that gives the sensor probe superior tolerance against noise and drift. In our previous study [36] the differential sensor signals settled down to constant values immediately after sample solutions with high buffering capacities were suctioned in. Meanwhile, an overshoot occurred when we measured diluted solutions with low buffering capacities (β < 0.5 mM/pH), which increased both the settling time and measurement error. From these results, we surmised that the overshoot was caused by an LJP formed between the sample solution and BLS. We therefore wanted to construct a simple method that allowed us to shorten the settling time and decrease the measurement error with diluted sample solutions.

In the present study we focused on the control of the LJ at the small border region in the sensor probe in order to improve the measurement performance. The active vibration of the border region to mix the two solutions resulted in an immediate settling of the overshoot after the sample solution was suctioned in, which in turn improved the measurement accuracy. We call this approach the “turbulent method” and contrarily call the alternative, the approach we did not apply, the “steady method”. We created a response curve by plotting the variable concentrations of KCl in the sample solution and BLS. The measurement error was almost zero when the KCl concentrations in the sample solution and BLS were equal, and increased in step with the KCl concentration differential between the two solutions. The turbulent method shortened the settling time to 15 s and decreased the measurement error to 0.060 pH. The averaged absolute value of the measurement error correspondingly decreased to 14% of that measured by the steady method, even with solutions with low buffering capacities (β ≈ 0.06 mM/pH). We expect that the method we report here will be applied to potentiometry and various other measurement methods compromised by measurement error caused by the LJP.

## 2. Experimental Section

### 2.1. Sensor Probe and Automated pH Measurement System

**Figure 1:**
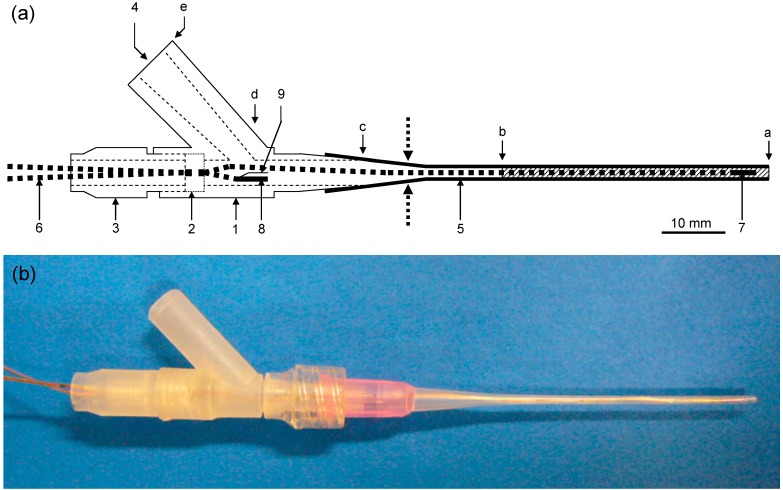
(**a**) A schematic representation and (**b**) view of the flow-through-type differential pH sensor probe. (a) 1, pH-sensor probe body; 2, O-ring; 3, screw to press the O-ring; 4, baseline solution (BLS) inlet; 5, Argyle Medicut Cannula; 6, lead wires; 7, measurement pH-FET; 8, reference pH-FET; 9, pseudo reference electrode; Hatch, suctioned sample solution. Inner volume of the probe (μL); a–b, sample suction volume = 26; a–c, 105; a–d, 242; a–e, 393; Dotted arrows, cut position for broadening the mouth of the cannula.

The flow-through-type differential pH sensor probe (Figure 1) used in this work is identical to that reported in our previous paper [35,36,37]. The sensor probe consists of one measurement pH-FET sensor, one reference pH-FET sensor, and one Ag/AgCl electrode. Each pH-FET measures 0.20 mm in thickness, 0.45 mm in width, and 5.5 mm in length. The measurement pH-FET is fitted inside a tube (cannula; Medicut^TM^ Cannula, Argyle, Tokyo, Japan) with an inner diameter of 0.89 mm and placed at the mouth of the probe. The reference pH-FET and a pseudo reference electrode (Ag/AgCl wire) are placed near the inlet of the BLS. The BLS is kept inside the sensor probe at all times, except when the actual measurement is performed. The sensor probe suctions in the sample solution (26 μL) from the top of the cannula to immerse the measurement pH-FET.

Measurements were performed by an automated pH measurement system (Auto-pH) [36]. The Auto-pH system washes and calibrates the sensor probe and measures up to 96 samples automatically and with high-reproducibility. The system consists of the sensor probe described above, a peristaltic pump (Minipuls 3, GILSON, Inc., Middleton, WI, USA), an XYZ stage (XA-S3, SUS Corp., Shizuoka, Japan), an overflow-type drain bottle, a 96-well microplate (NUNC, Rochester, NY, USA), a circuit for operating the two pH-FETs, and a PC. A 100 μL volume of the solution was poured into each well for measurement and calibration.

The total operation time for a single well was 132 s. This consisted of a washing period (60 s), 1st moving period (6 s), measuring period (60 s), and 2nd moving period (6 s).

The source potentials of the pH-FETs are expressed as:

ΔΔV = ΔV(1) – ΔV(0) = Vm(1) – Vr(1) – [Vm(0) – Vr(0)] = Vm(1) – Vm(0) – [Vr(1) – Vr(0)]
(1)

where Vm and Vr are the source potentials for the measurement and reference pH-FETs, respectively. Vm and Vr are measured using an Ag/AgCl pseudo reference electrode as a standard. The differential potential ΔV is defined as Vm – Vr at the same time, so the potential of the Ag/AgCl pseudo reference electrode is canceled out. ΔV(0) and ΔV(1) are the ΔV values in the final second of the washing period and the final second of the measurement period, respectively. ΔΔV is defined as the difference in the source potential of the measurement pH-FET, because Vr(1) – Vr(0) equals zero due to the remnant BLS inside the probe during measurement. The pH of the sample solution is calculated from the measured ΔΔV by the following equation:

pH = pH_0_ + ΔΔV/k (2)

where pH_0_ and k are ideally equal to the pH of the BLS and the pH sensitivity of the measurement pH-FET, respectively. Calibration using three standard solutions with pH values of 4.01, 6.86, and 9.18 yielded two constants, pH_0_ = 6.464 ± 0.003 and k = 54.3 ± 0.1 mV/pH.

### 2.2. Vibration and Mixing at the Liquid Junction during Measurement and Evaluation of Their Effect

An LJ between the sample solution and BLS forms in the flow-through-type sensor probe during measurement (Figure 1a, point “b”). Two solutions fed together into a tube will not mix if the tube is narrow and has a low Reynolds number (Re). If the two solutions have different electrolyte concentrations, an LJP will form between them and cause measurement error. Because the LJP forms in response to a separation of the electric charge, it will disappear if some means are used to break down the charge separation. By breaking the charge separation, we can therefore improve the measuring accuracy. Our strategy for achieving this was to mix the two solutions by forcibly vibrating the border region between them.

A sudden fluctuation of the flow velocity was required to mix the two solutions in the narrow tube (0.89 mm inner diameter) of the sensor probe. Hence, the pump tube (3.2 mm outer diameter, 1.15 mm inner diameter) was manually pushed and released at one point between the pump and probe twice every second (2 Hz). The solution in the sensor probe, which had a tenfold smaller volume than the sample solution (26 μL), was moved backward and forward by a volume of about 2 μL at the border region. The response time was unaffected by the movement volume but was shortened in proportion to the frequency of the vibration. We chose the 2 Hz frequency because it was the highest frequency applied in the present method. As previously mentioned, we call this approach and the approach without pushing and releasing the “turbulent method” and “steady method”, respectively.

We evaluated the effect of vibration and mixing on the LJ during the measurement by comparing the response curves, that is, the plots of the changes in the differential source potentials of the pH-FETs over time, between the steady method and turbulent method. At t = 0, the sensor probe read the differential source potential of the measurement and reference pH-FETs for the BLS, ΔV(0). Next, the probe was moved from the drain bottle to a sample well (from t = 0 to 6 s), the sample solution was suctioned into the probe (from t = 6 to 9 s), and the pump was shut off (from t = 9 to 66 s). In the measurements conducted by the turbulent method, the BLS was moved backward and forward to generate turbulence after the sample solution was suctioned in (from t = 9 to 66 s).

The settling time was defined as the time passed from the end of suction (t = 9 s) until the pH value reached less than ±1% of the final value (t = 66 s) [38]. The measurement error was defined as the difference between the final value and the authentic value measured by the glass electrode (Table 1). The overshoot, the difference between the value at the end of suction (t = 9 s) and the end of measurement (t = 66 s), was evaluated in the measurements performed by the turbulent method. For convenience, this paper reports all of the potential changes measured in mV units as pH units, after conversion by a calibration equation (Equation (2)). The response curves (described later) only show the responses for Solutions #1 to 4, as Solution # 3 was used as the BLS.

**Table 1:** Sample solutions (#1–4) and baseline solutions (#2–4).

Solution #	[KCI] (mM)	[Na_2_HPO_4_] (mM)	[KH_2_PO_4_] (mM)	Buffer Capacity (mM/pH)	pH after Air Bubbling
#1	0	0.061	0.017	0.064	6.625
#2	12.5	0.061	0.017	0.058	6.625
#3	50.0	0.061	0.017	0.065	6.625
#4	200.0	0.061	0.017	0.059	6.549

### 2.3. Solutions, Buffer Capacities, and Authentic pH Values

To determine how the ion concentrations of the solutions affected the overshoot potential and measurement errors, solutions with KCl concentrations of 0, 12.5, 50.0, and 200.0 mM were prepared by diluting 7.41 pH standard solutions 500 times with distilled water and adding the required amounts of KCl (Table 1). The buffer capacities and authentic pH values of the four solutions were almost the same. In fact, the only value found to vary between them was the KCl concentration.

The authentic pH values of the solutions were measured by a pH meter (pH/Ion Meter F-53, Horiba, Kyoto, Japan) and glass electrode (Type 9611, Horiba) under a stirring condition in a bottle. Air was bubbled into the solutions in amounts sufficient to prevent pH changes due to the atmospheric CO_2_. The pH values of the solutions shifted from weakly alkaline to weakly acidic during the air-bubbling. To allow for the very slow response of the glass electrode, the pH measurements were read after the value had plateaued, which in most cases was after at least 20 min. For this experiment we defined the buffer capacity as the quantity of strong acid that had to be added to change the pH of 1 liter of solution by 1 pH unit. The 10 mM HCl solution was added into 20 mL of each solution until the glass electrode pH measurement was reduced by 1.0. All reagents were obtained from Wako (Osaka, Japan). All measurements in this study were performed at room temperature (23–25 °C).

Solutions #2 to 4 were used as both sample solutions and BLS’s, while Solution #1 was used only as a sample solution. Solution #1 was found to be unsuitble for use as a BLS, as the measurement pH-FET generated remarkably increased levels of measurement noise when a BLS with a KCl concentration of less than 5 mM was used. When Solution #1 was used as the BLS, for example, the measurement noise generated by the measurement pH-FET was as large as 0.2–0.3 pH, far higher than the approximately 0.002 pH generated by the reference pH-FET.

### 2.4. Verification of the Mechanism Producing the Liquid Junction Potential

To elucidate where in the probe the liquid junction potential was formed, the reference pH-FET was moved stepwise upward from the mouth of the probe while the measurement pH-FET was kept fixed at the mouth. The mouth of the cannula was broadened for this experiment by making an incision at the arrowed position, as the original cannula shown in Figure 1 was too thin to accommodate both of the pH-FETs. The position of the reference pH-FET was moved upward in steps of 5, 10, 20, and 30 mm. The measurements were carried out at each position by the same pumping program described in Section 2.1. This experiment was performed using Solution #1 as the sample solution and Solution #4 as the BLS.

## 3. Results and Discussion

### 3.1. A newly Developed Method for Decreasing the Liquid Junction Potential

The object of our research was to develop a simple method to decrease the measurement error of a flow-through-type differential sensor probe composed of two pH-FETs by solving the heretofore inescapable LJP problem. The LJP has been especially problematic for the measurement of diluted sample solutions such as those with low buffering capacities and low ion concentrations. To accomplish our objective, we sought a method to exert spatiotemporal control over the LJ in a tiny sensor probe based on microfluidics.

We found a simple method for converging the sensor signal to a stable value by vibrating the border region of the two solutions to mix the solutions. The turbulent method shortened the measurement (Section 3.2) and improved the measurement accuracy (Section 3.3) by suppressing the overshoot and shortening the settling time after the sample solution was suctioned in. To our knowledge, no other method to decrease the LJP via a mechanical action has ever been reported. The notion that the LJP can be decreased via control of the LJ formed in a tiny sensor probe has attracted a great deal of attention in the field of potentiometry. The device used for the present method is unsusceptible to contamination caused by the leakage of saturated KCl, a problem that often occurs in the glass electrode method. The device is also more versatile than water-immiscible ionic liquid salt bridges, as it can be used to measure diluted solutions containing hydrophobic ions. In the future we expect to improve the sensor probe used in this work by optimizing parameters such as the channel dimensions and measuring conditions (Section 3.6).

We evaluated the effect of the turbulent method from the sensor signal directly. The sensor probe responded quickly (msec order) because the pH-sensitive region of the pH-FET used in the instrument was coated with Ta_2_O_5_ [39,40,41,42,43]. This rapid response made it possible to monitor the transient characteristics of the measurement directly from the sensor signal. The use of Auto-pH [36] enabled data acquisition with high reproducibility, as the system washed the sensor probe, suctioned in the sample solution, and performed all other actions rapidly and automatically.

### 3.2. Effects of Vibration and Mixing on the Liquid Junction

**Figure 2:**
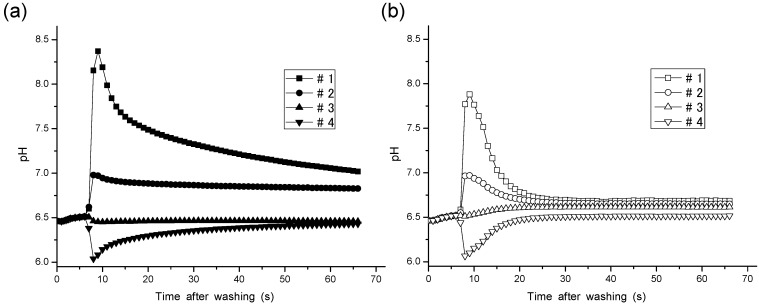
Response curves for Solutions #1–4 using Solution #3 as a BLS, by the steady method (**a**) and turbulent method (**b**).

Figure 2 shows response curves measured by the steady method (a) and turbulent method (b). The settling time was largely shortened by the application of the turbulent method in each sample solution (#1–4). The turbulent method shortened the settling time to 15 s. The pH values measured and averaged measurement errors of the two methods are compared in Table 2. The averaged absolute values of the measurement errors of the steady method and turbulent method were 0.221 and 0.032, respectively. Hence, the measurement error of the turbulent method was reduced to 14% of that of the steady method.

**Table 2:** Comparison of the measured pH values and measurement errors in the four solutions (#1–4), using Solution #3 as a BLS obtained from the two approaches.

Method	pH Values/Errors	#1	#2	#3	#4	Average of Absolute Error
Steady method	pH (Auto-pH)	7.019	6.828	6.458	6.429	-
	Measurement Error	0.394	0.2.3	−0.617	−0.120	0.221
Thrbulent method	pH (Auto-pH)	6.684	6.649	6.615	6.517	-
	Measurement Error	0.060	0.025	−0.032	−0.032	0.0318

The settling time of the diluted solution was reduced to 15 s in our experiments. The measurement of diluted solutions using conventional glass electrodes takes over 10 min to reach the measurement value plateau. From this perspective, the turbulent method applied with the flow-through-type differential pH sensor probe provides great advantages by shortening the measurement time [35,36,37].

### 3.3. Dependency of the Measurement Error on the Ion Concentrations

**Figure 3:**
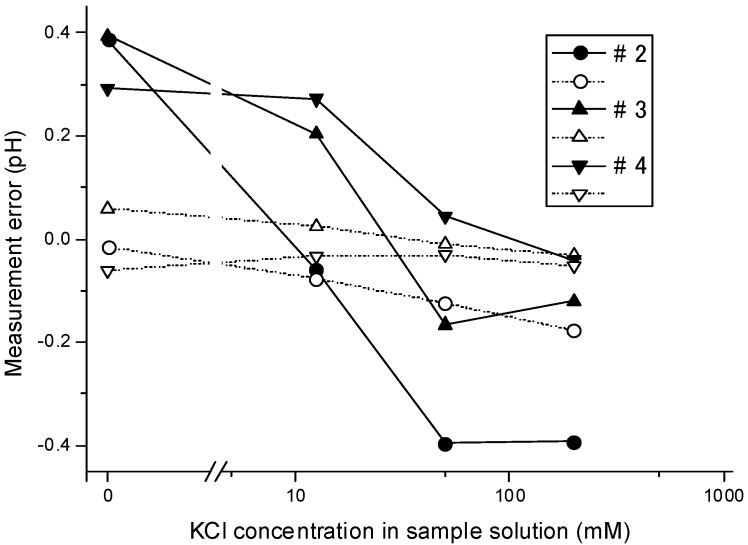
Dependency of measurement errors on the KCl concentrations in sample solutions measured using Solutions #2–4 as the BLS, by the steady method (solid line) and turbulent method (dotted line).

Figure 3 plots the measurement errors against the KCl concentration in the sample solutions. As the figure shows, a fairly large measurement error (from −0.4 to +0.4 pH) was observed in the steady method. The error in the turbulent method was far lower: In the measurements using BLSs with KCl concentrations of 50 mM (#3) and 200 mM (#4), the measurement error had an absolute value of less than 0.060 pH.

Solution #1 was never used as a BLS because it generated noise, presumably via the impedance between the measurement pH and Ag/AgCl pseudo reference electrode. Compared with the reference pH-FET, the measurement pH-FET was set at a further distance from the Ag/AgCl pseudo reference electrode in the sensor probe (Figure 1a).

### 3.4. Dependency of the Overshoot on the Ion Concentration

**Figure 4:**
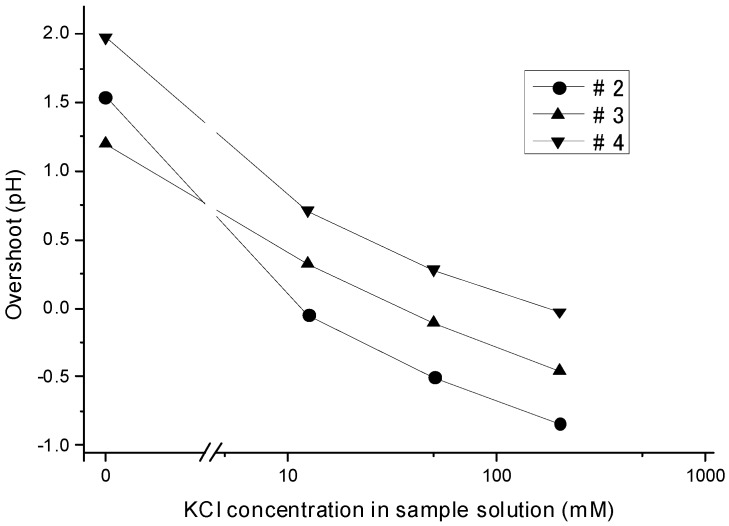
Dependency of overshoot on the KCl concentration in the sample solution using Solutions #2–4 as the BLS.

Figure 4 plots the degree of overshoot in the turbulent method against the KCl concentration in the sample solutions. The overshoot plotted in curve #3 in the figure, the plot for the measurements performed with the 50 mM KCl BLS, was obtained by subtracting the pH values at t = 66 from those at t = 9 in the response curves in Figure 2b. Curves #2 and #4 in the figure, the plots for the BLS with 12.5 and 200 mM KCl, were obtained by a similar procedure.

As the figure shows, an overshoot potential towards the higher pH direction appeared when the KCl concentration in the BLS exceeded that in the sample solution, and *vice versa*. Hypothetically, the overshoot should be exactly 0 pH when the KCl concentrations in the two solutions are the same. Yet for reasons that still elude us, small overshoots were observed in the actual experiments (−0.049, −0.105, and −0.027 pH for [KCl] = 12.5, 50, and 200 mM, respectively). The three lines in the KCl concentration range from 12.5 to 200 mM were almost parallel. The average slope of these three lines was 0.38 pH, as the KCl concentration in the sample solution decreased to 1/4. This slope corresponded to −0.63 pH (34 mV)/decade [KCl] in the sample solution.

### 3.5. Mechanism by Which the Liquid Junction Potential Was Produced

**Figure 5:**
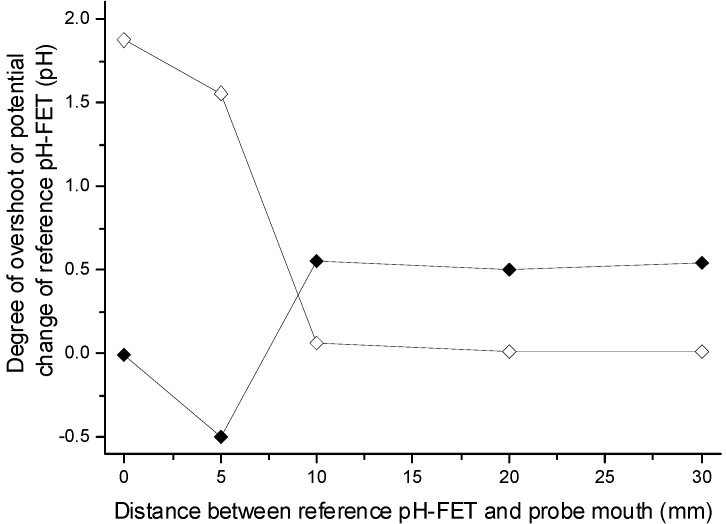
Effects of the distance of the reference pH-FET from the mouth of the probe on the degree of overshoot (**black**) and potential change of the reference pH-FET for the sample (**white**) just after sample suction. The measurement pH-FET was fixed at the mouth of the probe.

In Figure 5, the degree of overshoot observed and the potential change of the reference pH-FET just after sample suction are plotted against the distance (d_m-r_) between the broadened mouth and the reference pH-FET. When d_m-r_ was 0, that is, when the measurement and reference pH-FETs were both placed at the mouth of the cut cannula (see the dotted arrows in Figure 1), the two pH-FETs showed the same potential change after 26 μL of sample solution was suctioned into the probe, resulting in almost zero overshoot. When the reference pH-FET was moved to d_m-r_ = 10, 20, or 30 mm, a constant overshoot of about 0.5 pH was observed, as Figure 5 shows.

The potential of the reference pH-FET itself against the pseudo reference electrode changed by 1.9 pH (103 mV) at d_m-r_ = 0 on the sample suction. Solutions #1 and #4 had almost the same pH, but their KCl concentrations differed (0 and 200 mM, respectively). Therefore, the potential of the chloride ion-sensitive pseudo reference electrode (Ag/AgCl) was presumed to have changed. At d_m-r_ = 10, 20, 30 mm, the potential change of the reference pH-FET was almost zero. This suggests that the reference pH-FET remained inside the BLS both before and after the sample suction. In conclusion, Figure 5 seems to show that the overshoot potential is only observable when the reference electrode is placed above the suctioned sample solution. This, in turn, suggests that the overshoot potential is produced at the border region between the BLS and suctioned sample solution.

**Figure 6:**
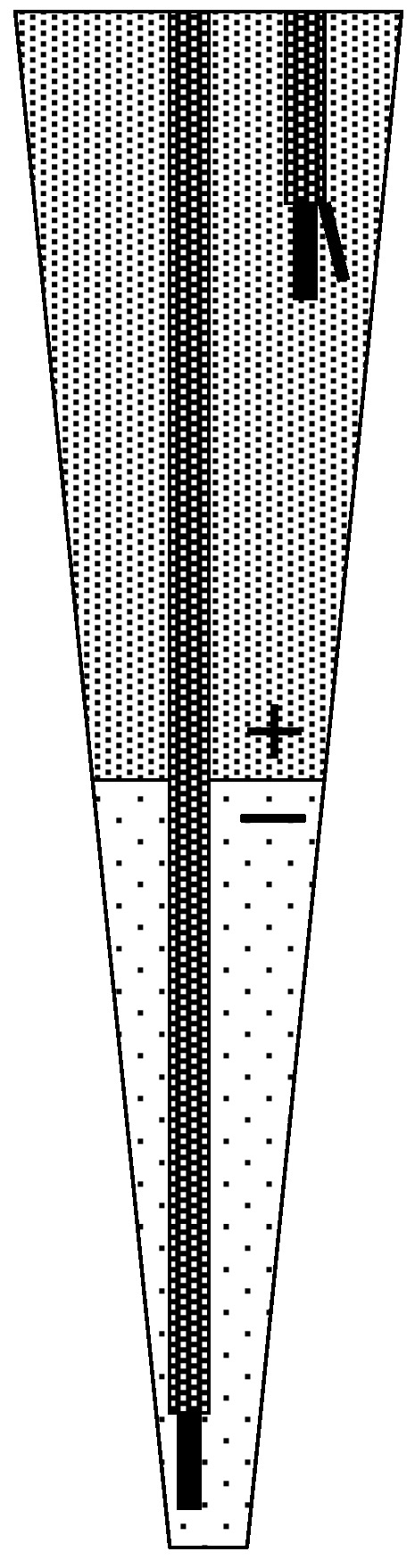
Estimated direction of the charge separation at the border region between the baseline and sample solutions, with the KCl concentration higher in the BLS higher than in the sample solution.

We estimate that the LJP is produced by the following mechanism. From Figure 4, we clearly see that the overshoot was caused by the difference between the KCl concentrations of the sample solution and BLS. Figure 5, meanwhile, suggests that this overshoot was produced in the border region between the BLS and suctioned sample solution. Figure 4 demonstrates that an overshoot potential toward the higher pH direction appeared when the KCl concentration in the BLS exceeded that in the sample solution. The higher pH direction was the direction in which the potential of the measurement pH-FET was negative relative to that of the reference pH-FET. Therefore, the charge separation schematically shown in Figure 6 in this case may have taken place at the border region, wherein the BLS side with the higher KCl concentration took a positive charge and the sample solution side with the lower KCl concentration took a negative charge. In contrast, an overshoot potential toward the lower pH direction appeared when the KCl concentration in the sample solution exceeded that in the BLS, and the charge separation was inverted. Whatever the case, the higher and lower KCl concentration sides are presumed to have taken a positive charge and negative charge, respectively.

### 3.6. Prospects for Miniaturizing the Device and Improving the Measurement Performance

The microfluidic technologies in the present sensor probe could be used not only to miniaturize the external form, but also to modify the fluid flows. This type of application would require an improved mixing profile and the leeway to dramatically reduce the LJP. Many technologies related to two-fluid mixing in microchannels have been described [34,44]. The present sensor probe has yet to fully optimize the mixing profile parameters such as the channel dimensions, flow velocity, and surface structures of the channel. The technologies described above would facilitate these improvements desired.

## 4. Conclusions

We have used microfluidics principles to improve pH measurement performance in a flow-through-type differential pH sensor probe composed of pH-FETs. Specifically, we exerted spatiotemporal control over the LJ of two solutions by vibrating the border region between the solutions and actively mixing them. The vibration and mixing were achieved simply by manually pushing and releasing the tube of the solution-delivery pump. By using of this method, the overshoot of the sensor signal caused by the LJP settled down immediately the sample solution was suctioned in, and the measurement error was reduced to only 0.060 pH even with a sample solution with a low buffering capacity or low ion concentration. The new method may be applied to many potentiometric measurement apparatuses using salt bridges (e.g., integrated glass electrodes) and is expected to improve the settling time, measurement accuracy, and other response properties. In the future we hope to understand the exact mechanism by which the active mixing eliminates the LJP in the micro scale region. A fuller understanding of the microfluidics phenomena at work may allow us to construct even smaller measurement devices with shorter measurement times and higher measurement accuracy.
